# Prevalence of type 2 diabetes mellitus and association of HbA1c with severity of coronary artery disease in patients presenting as non-diabetic acute coronary syndrome

**DOI:** 10.1186/s43044-020-00101-0

**Published:** 2020-09-29

**Authors:** Mohd Iqbal Dar, Jahangir Rashid Beig, Iqra Jan, Tariq Rashid Shah, Muzaffar Ali, Hilal A. Rather, Nisar A. Tramboo

**Affiliations:** 1 Department of Cardiology, SKIMS Soura, Srinagar, Jammu and Kashmir 190011 India; 2 Department of Immunology and Molecular Medicine, SKIMS Soura, Srinagar, Jammu and Kashmir 190011 India

**Keywords:** Acute coronary syndrome, Diabetes, Coronary angiography, HbA1c

## Abstract

**Background:**

Acute coronary syndrome (ACS) indicates the serious clinical manifestation of coronary artery disease (CAD) and is closely associated with cardiovascular prognosis in patients with ACS. This study was aimed to study the prevalence of type 2 diabetes mellitus (T2DM) and the relation of HbA1c with the severity of CAD in patients presenting as non-diabetic ACS. Diabetic status of the patients was assessed with fasting blood sugar (FBS) and HbA1c levels, and coronary artery disease burden was assessed by coronary angiography.

**Results:**

Out of 208 patients, 85.1% were males, and 14.9% were females; 73.56% cases were hypertensive. 80.77% of cases had STEMI, 17.79% had NSTEMI, and 1.44% had unstable angina. Out of 168 STEMI patients, 64.3% were thrombolysed, 21.42% presented late, 2.38% had contraindications to thrombolysis, and 11.9% underwent primary PCI. FBS in diabetic range was found in 44.23% of cases, impaired FBS in 36.54%, and 19.23% of patients had FBS in non-diabetic range. According to HbA1c, 41.8% were diabetic, 39.4% were pre-diabetic, and 18.8% were non-diabetic. A significant positive correlation was found between HbA1c and Gensini score and between HbA1c and the number of vessels involved.

**Conclusion:**

This study emphasises the importance of evaluating the presence of diabetes in patients presenting as non-diabetic acute coronary syndrome in developing countries. Acute coronary syndrome may be considered as one of the presentations of diabetes mellitus.

## Background

Acute coronary syndrome (ACS) indicates a serious clinical manifestation of coronary artery disease (CAD) and is the major cause of morbidity and mortality worldwide. The severity of coronary atherosclerosis is closely associated with cardiovascular prognosis in patients with ACS [1]. Consequently, the prediction and diagnosis of the extent of the coronary lesion in ACS are important for the clinical management of this disease. Coronary artery disease (CAD) has emerged as a leading cause of morbidity and mortality world over and more so in developing countries like India. Major risk factors for coronary artery disease include smoking, hypertension, diabetes mellitus, dyslipidemia, family history of CAD, and obesity [2].

Diabetes mellitus (DM) is one of the important and independent predictors of mortality in CAD. In addition to fasting blood sugar levels, one important way of assessing glycaemic control in diabetic patients is by estimating the blood levels of glycated haemoglobin (HbA1c) [3]. Glycated haemoglobin is produced by non-enzymatic condensation of glucose molecules with free amino groups on the globulin component of haemoglobin. The higher the prevailing ambient levels of blood glucose, the higher will be the level of glycated haemoglobin [4].

This study was aimed to assess the prevalence of type 2 diabetes mellitus (T2DM) and the relation of HbA1c with the severity of coronary artery disease in patients presenting as non-diabetic acute coronary syndrome

## Methods

This prospective observational cross-sectional study was conducted in the Department of Cardiology of our institute which is the lone tertiary care centre of our state. The acceptable sample size for this study at confidence interval of 99% and margin of error 10% was 166.

### Inclusion criteria

Patients presenting as non-diabetic acute coronary syndrome and admitted in the Department of Cardiology of our centre were included in this study.

### Exclusion criteria

Patients who denied consent for the procedure, known diabetics, patients with known coronary artery disease and history of a serious reaction to the contrast medium, renal transplantation, or end-stage renal disease necessitating dialysis were excluded from the study.

### Pre-procedural assessment

A detailed and comprehensive evaluation of each patient was done in the study which included relevant history taking, general physical and cardiac examination, complete baseline investigations, fasting blood sugar levels, serum HbA1c levels, and echocardiography. Blood HbA1c levels were assessed at a single laboratory to standardise the test value using high-performance liquid chromatography method (using BIORAD machine).

Patients were diagnosed as diabetics, pre-diabetes, and non-diabetics depending upon fasting blood sugar and serum HbA1c according to current American Diabetic Association (ADA) criteria. Patients having fasting blood sugar level ≥ 126 mg/dl or HbA1c ≥ 6.4% were diagnosed as diabetics. Patients with FBG between 100 and 125 mg/dl and HbA1c of 5.7 to 6.4% were diagnosed as pre-diabetes, and patients with fasting blood sugar levels < 100 mg/dl and HbA1c levels below 5.7% were diagnosed as non-diabetics [5].

### Coronary angiography and evaluation of coronary artery disease burden

Coronary angiography was performed by two dedicated interventionists by radial or femoral route depending on the operator’s discretion. The assessment of the burden of coronary artery disease on coronary angiography was done by two senior-most cardiologists to maintain uniformity and to reduce the inter-observer variability in reading the coronary angiogram. The assessment of CAD burden was done by calculating the Gensini score.

Gensini score is one of the multiple methods of assessing the burden of coronary artery disease. This scoring system was first published by Goffredo G. Gensini in 1983 in The American Journal of Cardiology. In this system, a numerical value is allocated to each degree of stenosis, and this provides a detailed assessment of coronary artery disease (CAD) and does not ignore even very trivial lesions in coronary arteries. Gensini score is one of the widely used coronary scoring systems. It also correlates well with average plaque burden and plaque area by IVUS as demonstrated by subsequent studies [6]. Gensini score calculates the burden of coronary artery disease depending upon the severity of the coronary lesion, its location, and area at risk with the location and extent of the coronary lesion.

**Fig. 1:**
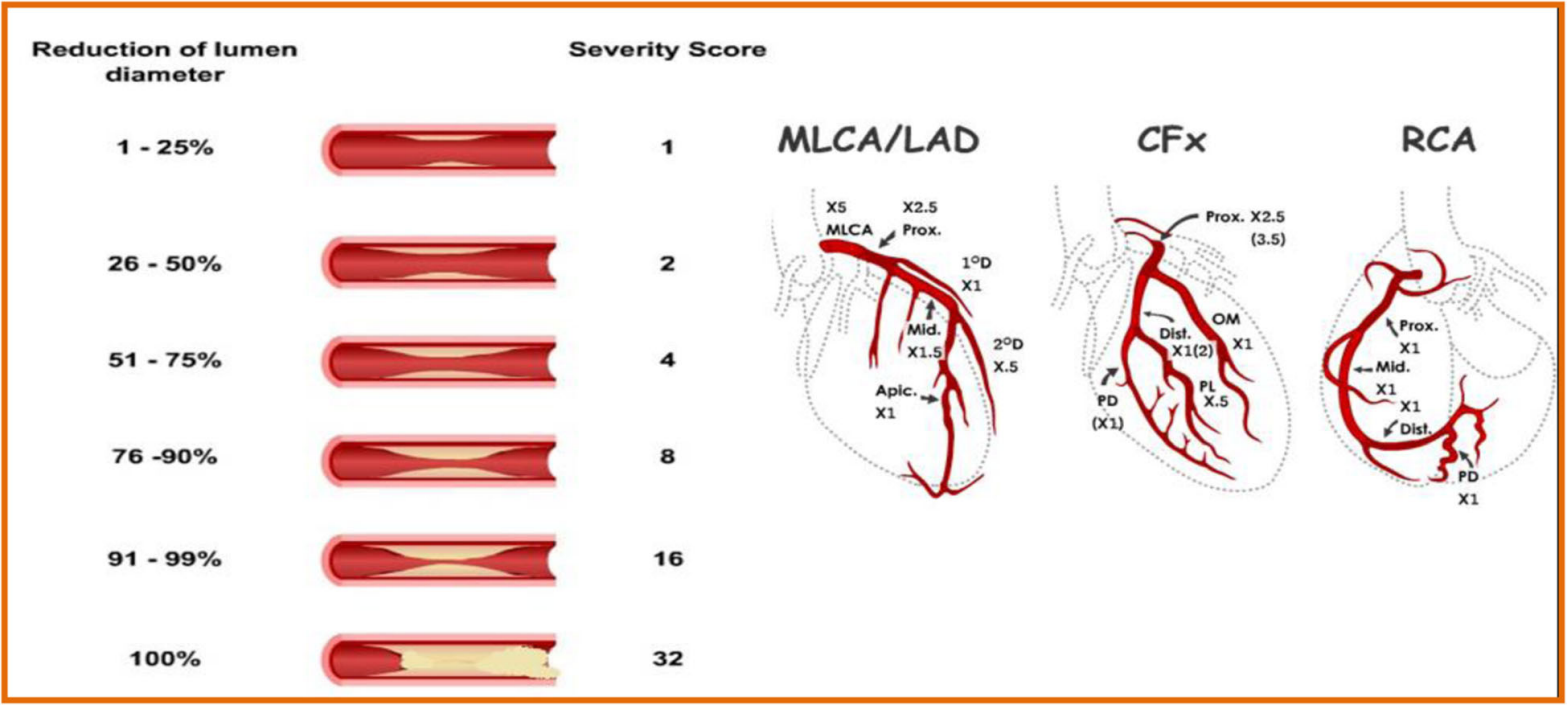
Calculation of Gensini score [7]

Gensini score (Fig. 1) grades narrowing of the lumen as follows: 1 = 1–25% occlusion; 2 = 26–50% occlusion; 4 = 51–75% occlusion; 8 = 76–90% occlusion; 16 = 91–99% occlusion; and 32 = total occlusion. This is based on the fact that with each step in the 25–50–75–90–99–100% diameter reduction progression, the impact on flow doubles in accordance with Poiseuille’s law. As a result, severity scores for lesions in this progression were assigned the values 1–2–4–8–16–32. This score is multiplied by a factor accounting for the importance of the lesion position in the coronary arterial tree, such as 5 for LM, 2.5 for proximal LAD, and 1 for proximal RCA. The severity of the disease is expressed as the sum of the scores for individual lesions [8].

### Statistical analysis

The recorded data was exported to SPSS version 23.0 after being compiled in Microsoft excel. Continuous variables were expressed as mean ± SD, and categorical variables were expressed as frequencies and percentages. Student’s independent *t* test was employed for comparing continuous variables. To determine the correlation of HbA1c with Gensini score and the number of vessels involved, Karl Pearson’s correlation coefficient was applied. A *p* value of less than 0.05 was considered statistically significant, and all *p* values were two-tailed.

## Results

At our centre, during the 2 ½ years of the study period from May 2017 to October 2019, a total of 1014 consecutive patients were admitted as acute coronary syndrome. Seven hundred and seventy patients (75.6%) were known diabetics on various antidiabetic therapies, and the rest of 244 patients (24.4%) presented as non-diabetic patients with acute coronary syndrome. Out of 244 patients, 208 patients were enrolled after taking in consideration the inclusion and exclusion criteria. Out of 36 patients excluded from the study, 20 patients had denied consent for participation in the study, 6 patients had a history of contrast allergy, 6 patients were having known coronary artery disease, and 4 patients were having end-stage renal disease. The mean age of the patients was 55.8 ± 9.0 years with a minimum age of 33 years and the maximum age of 76 years. Demographic characteristics and risk factors of the study population are shown in Table 1.

**Table 1:** Demographic characteristic and risk factors

			Number of patients (%)
Sex	Male		177 (85.1)
	Female		31 (14.9)
	Total		208 (100)
Hypertension	Male		140 (79.09)
	Female		13 (41.93)
	Total		153 (73.6)
Smoker	Male		155 (87.5)
	Female		14 (45.16)
	Total		169 (81.3)
Dyslipidemia	Male		152 (85.87)
	Female		25 (80.64)
	Total		177 (85.1)
Body mass index (BMI)	Male	18.5–24.9	142 (80.02)
		25–29.9	24 (13.5)
		30–35	11 (6.2)
	Female	18.5–24.9	18 (58.08)
		25–29.9	6 (19.35)
		30–35	7 (22.58)
	Total	≥ 25	50 (24.4)

ST-elevation myocardial infarction was the most common ACS in this study. One hundred and sixty-eight (80.77%) of patients had STEMI, 37 (17.79%) had NSTEMI, and only 3 (1.44%) patients presented as unstable angina. Anterior wall MI was the most common presentation of STEMI with 84 (50%) out of 168 STEMI of cases having this diagnosis on presentation followed by an inferior wall in 74 (46.43%) and anterolateral wall in 6 (3.57%) cases.

The different therapeutic interventions received by 168 STEMI and the thrombolytic drugs used are shown in Table 2. Out of 4 patients with contraindications to thrombolysis, 2 patients had developed myocardial infarction post-operatively, one patient had previous history of hemorrhagic stroke, and one patient had a history of recent (2 months) ischemic stroke.

**Table 2:** Therapeutic interventions and thrombolytic drugs used in STEMI patient population

		*N* (%)
Therapeutic intervention	Thrombolysis	108 (64.30)
	Primary PCI	20 (11.90)
	Late presentation	36 (21.42)
	Thrombolysis contraindicated	04 (2.38)
	Total	168 (100)
Thrombolytic drug	Streptokinase	49 (45.37)
	Tenecteplase	41 (37.97)
	Reteplase	18 (16.66)
	Total	108 (100)

**Fig. 2:**
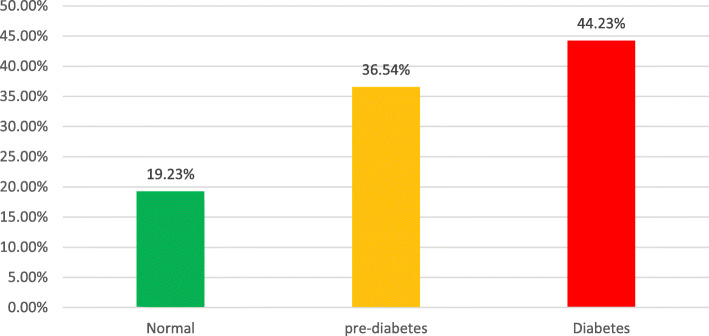
Fasting blood sugar levels

Fasting blood sugar levels of the study population is given in (Fig. 2). Majority of the patients were having fasting blood sugar levels in the pre-diabetic or diabetic range. Less than 1/5 of the patients had normal fasting blood sugar levels. Additionally, 9 (4.23%) patients in the diabetic group presented with diabetic ketoacidosis.

**Fig. 3:**
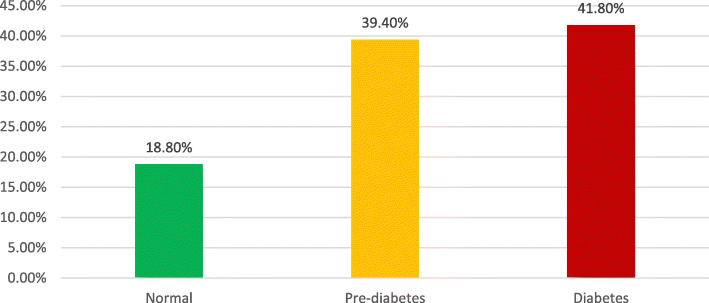
HbA1c levels of the study population

HbA1c levels of the study population are shown in Fig. 3. The study showed 41.8% of cases HbA1c levels in the diabetic range, 39.4% of the cases HbA1c in pre-diabetics, and only 18.8% of the cases were having non-diabetic levels of HbA1c. On the basis of HbA1c and subsequent normalisation of blood sugars, 5 (2.40%) patients were found to have stress hyperglycemia with HbA1c in pre-diabetic range, and 1 (0.48%) patient with normal initial blood sugars was found to be pre-diabetic.

On coronary angiography, single-vessel disease was noticed in 105 (50.48%), double-vessel in 78 (37.5%), and triple-vessel in 25 (12.08%) cases. Right coronary artery (RCA) was most commonly involved with 63.41% cases having RCA disease, left anterior descending (LAD) coronary involvement was seen in 55.29%, and left circumflex (LCX) in 36.54% cases.

The burden of coronary disease as assessed by Gensini score varied widely with a minimum score of 8 and a maximum score of 48 as shown in Table 3. More than half of patients (57.69%) had Gensini score more than 20 indicating a high coronary atherosclerotic burden.

**Table 3:** Gensini score of the study

	< 20	> 20	Total
*N* (%)	88 (42.3)	120 (57.69)	208 (100)

**Fig. 4:**
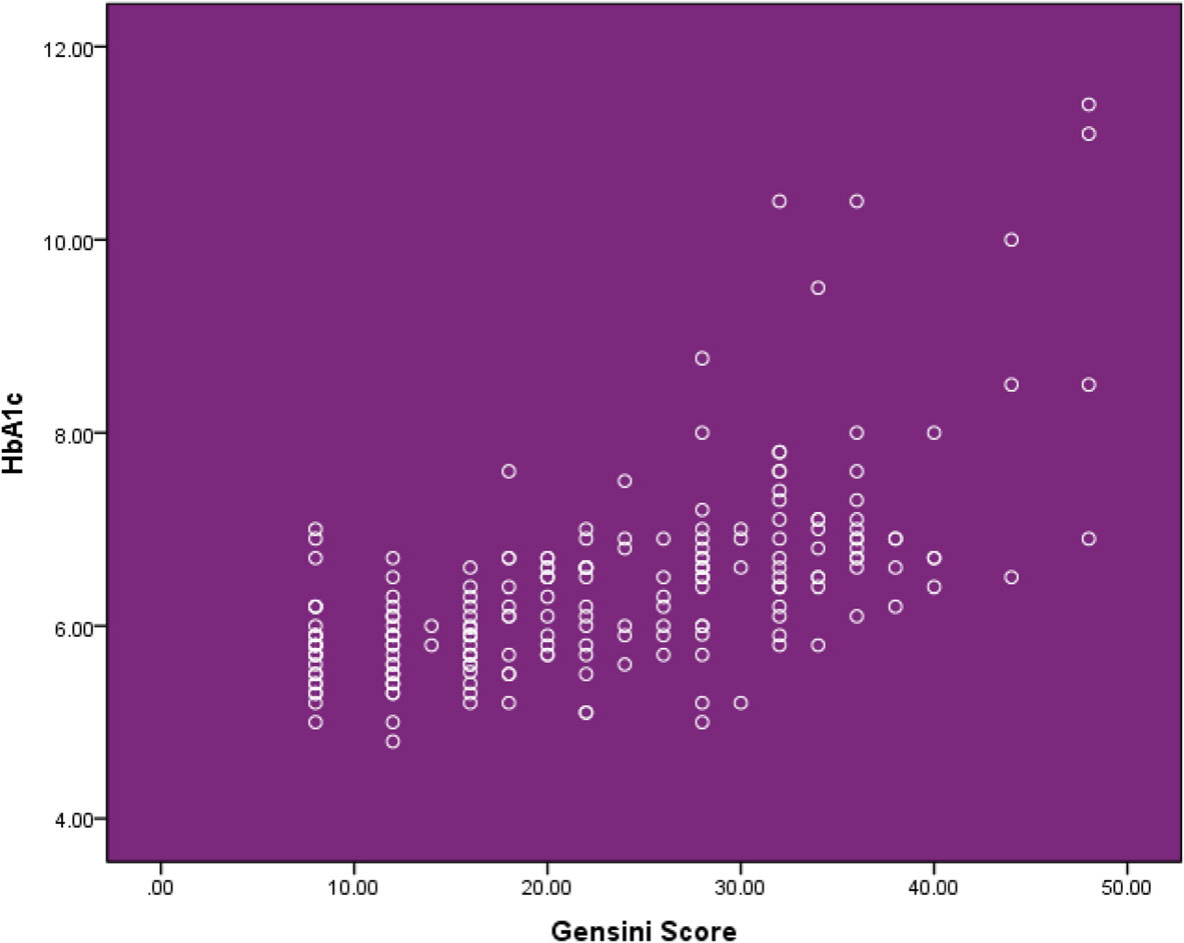
Scatter plot showing correlation between HbA1c and Gensini score of the study

Bivariate two-tailed correlation analysis showed a significant positive correlation between HbA1c and Gensini score with Pearson correlation value of 0.586 and *p* value < 0.001. This correlation is further depicted by scatter plot in Fig. 4.

A significant positive correlation was also seen by bivariate two-tailed correlation analysis between HbA1c and number of coronary vessels involved with Pearson correlation value of 0.566 and *p* value < 0.001.

## Discussion

Coronary artery disease continues to contribute significantly to the strain on healthcare resources world over. The major risk factors contributing to the development of CAD remains same throughout the world; however, there are differences in the contribution of the individual risk factor for the development of CAD in developing and developed world [9].

The mean age of patients in our study was 55.8 ± 9 years with 72.12% of patients between the ages of 40 to 60 years. The mean age of the patients in our study was lower, and the majority of patients were younger than that in other studies [10, 11]. This data was consistent with previous studies done in India which show that CAD presents a decade early in Indian patients [12–16]. A strong male preponderance was noted with male: female ratio of 5.6:1 which was consistent with the other studies conducted earlier at this centre and the rest of the country [17–19]. Majority of patients were having BMI in the normal range. More female patients were overweight or having grade 1 obesity than the male counterparts likely owning to the relatively sedentary lifestyle in females [17, 20]. Dyslipidemia was the most common risk factor associated with this study group of patients. Dyslipidemia prevalence is significantly higher than that reported in other studies in which no segregation of diabetic and non-diabetic patients presenting as ACS was done [19, 21].

Smoking was the second most prevalent risk factor associated in this study. 81.25% of cases had an active smoking history. It was more common in males than in females. Smoking prevalence is significantly higher in this study than other studies from across the country and abroad because the general population prevalence of smoking in our state is twice the national average as reported by the Global Adult Tobacco Survey-2 (GATS-2) 2016–2017 [15, 22, 23]. Hypertension was the third most common risk factor identified, and the prevalence of hypertension in males was nearly twice that of females. This male preponderance may be due to non-segregated nature of other studies.

ST-elevation myocardial infarction (STEMI) was the most common presentation of acute coronary syndrome (ACS). This data reinforces the fact that ACS is the most common presentation of coronary diseases in developing countries than developed countries because of lack of awareness and infrastructure for picking up patients early like stable chronic angina [24, 25].

The percentage of patients presenting in the therapeutic window for thrombolysis in our study was lower than the national average of 80% [26] mainly because our state is a hilly state with underdeveloped road connectivity, majority of the population resides far from urban centres, and delay in recognition of the medical condition, though the efforts are ongoing to develop infrastructure for implementing the pharmoco-invasive strategy involving early recognition of the myocardial infarction, encouraging thrombolysis at peripheral centres wherever indicated and urgent referral to PCI-capable centre. Only 11.9% of patients received primary PCI as the therapeutic intervention which was consistent with the national data from other studies from the developing world [27]. This is in sharp contrast to the scenario in developed countries like the USA where 70–80% of the patients with acute coronary syndrome receive primary PCI due to the presence of a large number of PCI-capable centres throughout the country and majority of the population residing within 30–60 min away from these centres [28]. The reason for low primary PCI procedure in developing countries like ours are multifactorial but the main reasons being lack of adequate number of PCI-capable centres within the quick reach of the majority of the population resulting in loss of precious time for intervention, lack of health insurance cover, low socioeconomic status, and lack of awareness [19].

Older non-fibrin-specific drug streptokinase was the most commonly used thrombolytic agent, and the use of never fibrin-specific drugs like tenecteplase and reteplase was low. The same reasons as for low primary PCI intervention contributed to lower penetrance of use of newer fibrin-specific thrombolytic drugs in this study. The incidence of thrombolytic drug-related complication was low within our study population, only 3 cases of minor anaphylaxis with streptokinase and only 2 cases of minor bleeding (1 with streptokinase and 1 with tenecteplase) which were managed conservatively. The probable reasons for lower drug-related complications in this study were younger age of the patients and less associated co-morbidities.

Fasting blood sugar levels were high in the majority of patients. Only 19.23% of the patients had normal (< 100 mg/dl) fasting blood sugar levels. Overall, 80.77% of patients in our study were either diabetic or were having impaired fasting blood sugars. This data is significantly higher than the data reported in other Indian and foreign studies [21, 29]. Similarly, the HbA1c levels confirmed the diagnosis of diabetes mellitus in over 40% of cases, and another 39.42% of patients were having HbA1c in the range of pre-diabetes. In total, more than 80% of cases were either diabetic or pre-diabetic, and only less than 20% of cases had HbA1c levels in the normal range. There are fewer studies in literature in this regard so far. The published literature from other countries like Japan put the incidence of diabetes in this group of patients at around 40 to 45% [11]. The data in our study shows double the prevalence of diabetes in these patients. Our data is consistent with the results obtained by Nanayakkara et al. [30] who showed 47% diabetic and 53% prediabetes in their study of similar patients. The higher incidence of diabetes in this study may be due to failure to detect diabetes early in the developing nations due to lack of community-based diabetes detection programme, ignoring the symptoms and lack of routine medical care or malignant nature of this disease along with other risk factors in Indian subcontinent population. In contrast to some studies, the fasting blood sugar levels and HbA1c levels correlated well with each other in the diagnosis of diabetes status in our study [21, 31].

Single vessel involvement was most common. Half of the patients had single-vessel disease and another 1/3 of patients was having double vessel involvement and only about 12% of cases had triple-vessel disease. This data was similar to earlier studies [22, 23]. The predominant reason for single-vessel involvement could be due to the presence of a large number of young patients with fewer comorbidities in this study cohort.

In this study, we used the Gensini score to estimate the disease burden of coronary vessels. Gensini score estimates the severity of atherosclerosis in coronary vessels very well and calculates the disease burden depending upon the severity of vessel stenosis, its location, and the area at risk with the lesion [6, 8]. Patients with Gensini scores of 20 or more are defined as having severe CAD, which was approximately equal to one stenosed lesion of 70% or more in the proximal left anterior descending artery [32]. The mean Gensini score in our study was 22.51 ± 10.37. Sixty percent of patients were having severe CAD with Gensini score of ≥ 20.

Bivariate two-tailed correlation analysis showed a significant positive correlation between HbA1c and Gensini score with Pearson correlation value of 0.586 and *p* value < 0.001. Similar results were obtained by correlation analysis between HbA1c and number of coronary vessels involved with Pearson correlation value of 0.566 and *p* value < 0.001. This data strongly supports the data from other emerging studies [10, 30, 33] strengthening the fact that besides diagnosing diabetes mellitus, HbA1c is strongly related with coronary artery disease and can be used as a marker of coronary artery disease and higher HbA1c levels predicting higher coronary artery disease burden.

### Study limitations

The major limitation of the study is that this is a single-centre study with a relatively small number of patients; a multicenter study with larger study cohort may be required to give a better estimate of study parameters.

## Conclusion

This study emphasises the importance of evaluating the presence of diabetes in patients presenting as non-diabetic acute coronary syndrome. In developing countries due to lack of early detection, the acute coronary syndrome may be considered as one of the presentations of diabetes mellitus. Furthermore, HbA1c could be considered as a marker of the presence and burden of coronary artery disease.

## Data Availability

All the data used in this study is available with the corresponding author.

## Abbreviations

ACS: Acute coronary syndrome
CAD: Coronary artery disease
T2DM: Type 2 diabetes mellitus
FBS: Fasting blood sugars
STEMI: ST-elevation myocardial infarction
NSTEMI: Non-ST-elevation myocardial infarction
IVUS: Intravascular ultrasonography
LAD: Left anterior descending
RCA: Right coronary artery
LCX: Left circumflex coronary artery
